# ROS homeostasis and metabolism: a dangerous liason in cancer cells

**DOI:** 10.1038/cddis.2016.105

**Published:** 2016-06-09

**Authors:** E Panieri, M M Santoro

**Affiliations:** 1 Department of Molecular Biotechnology and Health Sciences, Molecular Biotechnology Center, University of Turin, Turin, Italy; 2 Laboratory of Endothelial Molecular Biology, Vesalius Research Center, VIB, Leuven B-3000, Belgium; 3 Laboratory of Endothelial Molecular Biology, Vesalius Research Center, Department of Oncology, University of Leuven, Leuven B-3000, Belgium

## Abstract

Tumor cells harbor genetic alterations that promote a continuous and elevated production of reactive oxygen species. Whereas such oxidative stress conditions would be harmful to normal cells, they facilitate tumor growth in multiple ways by causing DNA damage and genomic instability, and ultimately, by reprogramming cancer cell metabolism. This review outlines the metabolic-dependent mechanisms that tumors engage in when faced with oxidative stress conditions that are critical for cancer progression by producing redox cofactors. In particular, we describe how the mitochondria has a key role in regulating the interplay between redox homeostasis and metabolism within tumor cells. Last, we will discuss the potential therapeutic use of agents that directly or indirectly block metabolism.

## Facts

- Deregulated redox homeostasis is a hallmark of cancer cells
- Increased ROS levels are able to promote tumor growth and malignant progression
- Increase antioxidant ability in malignant cells is a common feature
- Alteration of specific metabolic pathways in tumors is frequently found
- Tumors can be sensitized to chemotherapy and other antitumor treatment by disabling antioxidant defenses (NADPH and GSH) through metabolic inhibition

## Open Questions

- What are the redox-sensitive transducers that specifically promote signaling events in cancer cells?
- As metabolism can support the intracellular redox homeostasis by NADPH and GSH synthesis, what are the cancer-specific pathways/alterations that can be selectively targeted for therapeutic purposes?
- To what extent can the inhibition of antioxidant mechanisms be used to potentially enhance chemo/radiotherapy without inducing side toxicity on normal cells?
- Would it be possible to generate animal models that allow real-time detection of metabolic/redox intermediates with high spatial and temporal resolution during cancer progression?

**Figure 1:**
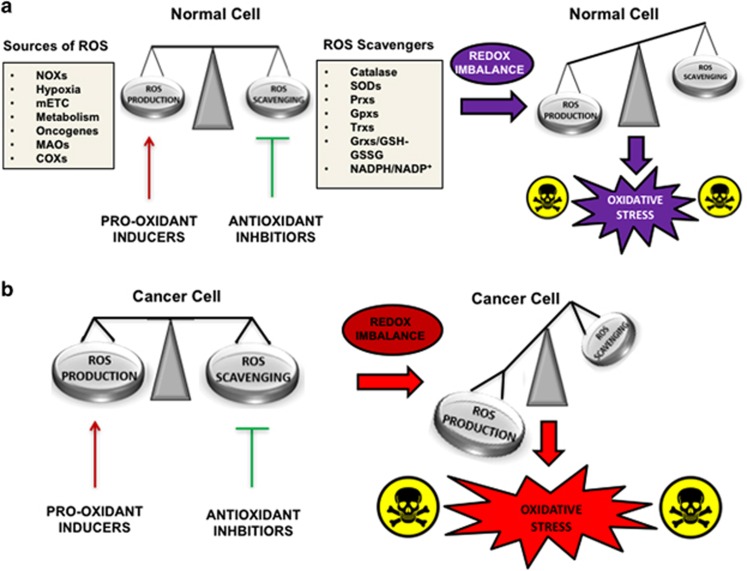
ROS sources and scavengers in the control of redox homeostasis in normal and cancer cells. (**a**) Normal cells keep constant ROS production and elimination to maintain a favorable redox balance. Disruption of redox homeostasis by co-treatment with ROS inducers and antioxidant inhibitors induces oxidative stress and variable levels of cell death. (**b**) Cancer cells exhibit higher steady-state levels of ROS counterbalanced by increased antioxidant capacity. The combined use of pro-oxidizing treatment and antioxidant inhibition is expected to cause severe oxidative stress and severe cytotoxicity

Cancer is one of the leading causes of death worldwide. Despite extensive research and considerable efforts for developing targeted therapies, many tumors are still characterized by poor prognosis and high mortality. For this reason, novel strategies to improve the outcome of patients suffering from aggressive or therapy-resistant malignancies are critically needed. Recent evidences indicate that altered redox balance and deregulated redox signaling, which are two common hallmarks of tumors, can be strongly implicated in malignant progression and resistance to treatment. It has been long postulated that cancer cells exhibit persistently high reactive oxygen species (ROS) levels as a consequence of genetic, metabolic and microenvironment-associated alterations. These are then compensated by an increased antioxidant ability from these cancer cells.^1^ Although seemingly paradoxical, this pro-oxidant shift can promote tumor growth by inducing DNA damage and genomic instability,^2^ which then activate an inflammatory response,^3^ stabilizing the hypoxia inducible factor-1^4^ and thus reprogramming metabolism.^5, 6^ Due to the selective pressure induced by sustained ROS production, cancer cells have developed an efficient mechanism of ROS detoxification that presents a selective advantage over and upholds its survival under pro-oxidizing conditions. Therefore, the dependency of cancer cells from their antioxidant systems represents a specific vulnerability that must be exploited to induce targeted cell death. This can be achieved by increasing oxidative stress above the toxicity threshold, sparing normal cells, which are characterized by having lower intracellular ROS levels (Figure 1).^7^ Due to their dualistic nature, ROS can act as ‘good' and ‘bad' molecules, and regulate cellular physiology or induce cytotoxicity depending on the magnitude, duration and site of their generation. Hence, strategies aimed at altering redox signaling events in tumor cells and intend to disable key antioxidant systems in the presence of ROS inducers might represent promising new anticancer treatments.^8^ Other research looks to the intimate connection between cellular metabolism and redox homeostasis. Their reciprocal relationship is used by cancer cells to generate building blocks for cellular growth or antioxidant power to prevent oxidative damage. By redirecting energetic substrates and metabolic intermediates into the biochemical pathways that generate key antioxidant molecules, malignant cells can directly support the mechanisms of ROS detoxification.^9, 10, 11^ Therapeutic manipulations aimed at disrupting this functional crosstalk or elevating the burden of oxidative stress in the presence of selective metabolic inhibitors might induce synthetic lethality or sensitize cancer cells in common therapies^8, 10, 12^

This review focuses on the adaptive mechanisms that tumors use to face oxidative stress conditions. We will discuss the role of ROS in regulating metabolism and progression in cancer cells. Last, we cover potential therapeutic usage of agents that directly or indirectly alter the tumor redox balance.

## ROS Homeostasis and Redox Cofactors in Normal and Tumor Cells

Redox homeostasis is an essential requisite for aerobic organisms. They are dependent on the balance between the rate and the magnitude of oxidant production and their elimination over time. ROS are short-lived molecules with unpaired electrons deriving from partially reduced molecular oxygen that are perpetually generated, transformed and eliminated in a variety of cellular processes including metabolism, proliferation, differentiation, immune system regulation and vascular remodeling. These oxygen-containing derivatives are comprised of free radicals such as the superoxide anion (O_2_^−^·) or the hydroxyl radical (OH•) as well as non-radical molecules including hypochlorous acid and hydrogen peroxide (H_2_O_2_).^13, 14^ Both exogenous and endogenous sources of ROS production have been extensively described over the past decade.^15^ The most biologically relevant are represented by the nicotinamide adenine dinucleotide phosphate (NADPH) oxidases, professional enzymes that catalyze the production of O_2_^−^· or H_2_O_2_ using NADPH as a reductant^16^ and the mitochondrial electron transport chain (mETC), wherein mainly complexes I and II generate O_2_^−^ through univalent reduction of molecular oxygen as a consequence of electron leakage during mitochondrial respiration using nicotinamide adenine dinucleotide (reduced form) (NADH) and FADH.^17, 18^

To keep a steady-state control over ROS production–detoxification and prevent the harmful effects, aerobic organisms have evolved a complex array of defensive systems. These systems comprise scavenging enzymes and several endogenous or dietary-assumed antioxidant agents that limit ROS accumulation. The most relevant antioxidant enzymes include (i) superoxide dismutases (SODs) that convert superoxide (O_2_.^−^) to less reactive H_2_O_2_, (ii) catalase that reduces H_2_O_2_ to water and molecular oxygen and (iii) glutathione peroxidases that eliminate H_2_O_2_ using reducing power derived from glutathione. Other important defensive mechanisms and mediators of redox signaling are represented by the peroxiredoxin, the thioredoxin (TRX) and the glutathione/glutaredoxin systems.^19, 20, 21, 22, 23, 24, 25, 26, 27, 28, 29, 30^ Due to intrinsic differences in the half-life, stability, chemical reactivity, cellular context, site and source of their generation (exogenous or endogenous), ROS can interact and modify different classes of biological macromolecules including DNA, lipids and proteins.^31, 32, 33, 34, 35, 36, 37^ The tight regulation of ROS production and detoxification over time and space represents the basis for the maintenance of an appropriate redox homeostasis and redox signaling events. Disruption of redox circuitries that control the turnover of ROS and the related redox signaling events has a profound impact on cellular physiology and in turn may lead to aberrant signaling, unrestrained accumulation of toxic byproducts, oxidative damage and cytotoxicity.^38^ Although low levels of ROS are believed to regulate redox signaling events, high doses are regarded as being responsible for cell toxicity.^31, 39, 40, 41, 42, 43, 44, 45, 46, 47, 48, 49^ It is also well accepted that the efficacy of many anticancer therapies, including chemotherapeutics and radiotherapy, largely depends on their ability to induce ROS accumulation and evoke cell toxicity and death.^14, 50, 51, 52, 53, 54, 55, 56^ The high levels of oxidative stress normally associated with malignant progression represent tumor-specific alteration that makes cancer cells vulnerable to further elevation of ROS and strongly dependent on their antioxidant defenses. Both extrinsic and intrinsic factors contribute to generate a persistent amount of high ROS levels in tumors. To prevent excessive oxidative stress and promote redox signaling, tumor cells strategically adjust multiple antioxidant enzymes and make extensive use of their metabolic pathways to provide an adequate supply of antioxidant molecules (such as reduced glutathione (GSH) and NADPH).^1^ On the basis of these observations, disabling the intrinsic antioxidant mechanisms by promoting ROS production has been conducted in several studies.^57, 58, 59, 60, 61, 62, 63^ Still, these studies highlight the growing interest in the scientific community towards therapeutic strategies that are aimed at disrupting the redox homeostasis of malignant cells. To identify new strategies and define redox regulation and ROS levels in the context of tumor progression, several laboratories have found success with new approaches on the basis of the metabolic blockade as anticancer treatment (Table 1). Such treatments not only impact tumor growth by starving the cell from specific metabolic pathways but also by changing the redox state within the tumor cell. Laboratories are achieving these with encouraging results trying to understand which metabolic pathway is directly related to redox homeostasis and how it achieves ROS production, or an antioxidant response, in cancer cells.

## Metabolic Pathways Involved in ROS Homeostasis in Cancer Cells

**Figure 2:**
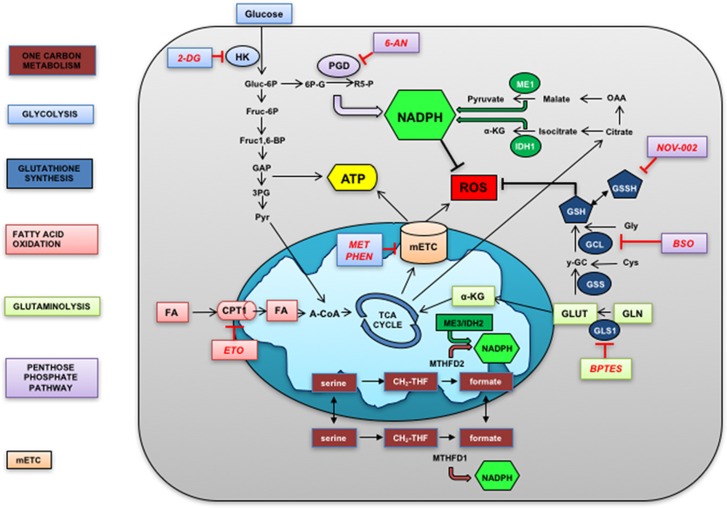
Cellular metabolic pathway involved in redox homeostasis. Schematic representation of central metabolic pathways described in the text and involved in redox homeostasis. Metabolic pathway in the cytosol and mitochondria are represented. Metabolites in lowercase, enzymes in uppercase and inhibitors in red. Color code indicates metabolic pathways. FA fatty acids; HK, hexokinases; ROS, reactive oxygen species; PGD, phosphogluconate dehydrogenase; ME1, malic enzyme; a-KG, alpha ketoglutarate

A growing body of evidence indicates that the malignant progression of tumors is characterized by the occurrence of multiple alterations where specific metabolic pathways are linked to the synthesis of essential building blocks (e.g., amino acids, lipids and nucleotides) fostering their uncontrolled growth. However, it is well recognized that part of the energetic substrates involved in these pathways can be also redirected into specific metabolic routes to generate not only antioxidant molecules (NADPH and GSH) but also redox cofactors (i.e., NADH and FADH) that can be readily used to maintain or restore an adequate redox homeostasis.^64, 65, 66, 67^ Increased attention has been dedicated to the intimate connection and reciprocal crosstalk between metabolism and redox balance of cancer cells, with a particular emphasis on the role of glycolysis, glutaminolysis, fatty acid oxidation (FAO), one-carbon metabolism and the pentose phosphate pathway (PPP).^68, 69, 70^ For this reason, it is important to analyze more in detail the major metabolic pathways that mainly control the redox homeostasis of cancer cells (Figure 2).

### Glycolysis

Glycolysis is an essential pathway occurring in the cytosol of mammalian cells through which glucose is transformed to pyruvate. Glucose is taken from the extracellular space by specific transporters (i.e., glucose transporters). Glucose is then converted to glucose-6-phosphate by hexokinase enzymes and enters into a series of ten enzyme-catalyzed reactions culminating in the generation of pyruvate, adenosine tris-phosphate (ATP) and reduced cofactors in the form of NADH.^71^ As already observed by Otto Warburg in the 1924, tumor cells exhibit a prevalent use of the glycolytic pathway regardless the presence of sufficient oxygen tension, a phenomenon known as Warburg effect.^64^ Several studies indicate that the pro-glycolytic shift caused by oncogene activation and loss of tumor suppressors represents a selective advantage for tumors by providing essential precursors for building the macromolecules required to sustain growth and proliferation.^72, 73^ As a matter of fact, therapeutic modulation of glucose metabolism and transport has been widely utilized as an effective anticancer strategy.^74, 75, 76, 77, 78, 79, 80^ It is now understood that glucose metabolism has an essential role in the control of redox homeostasis in tumors, as glycolytic intermediates can be shuttled into the metabolic pathways that directly or indirectly contribute to generate reducing equivalents, mainly PPP-derived NADPH or glutaminolysis-derived GSH. In this regard, a recent study showed that cancer cells exposed to glucose deprivation increase glucose metabolism to restrict the burden of ROS and prevent hydroperoxide-induced cell death.^81^ Also, inhibition of lactate dehydrogenase-A through the small-molecule FX11 impaired the malignant progression of lymphoma and pancreatic cancer xenografts by decreasing the intracellular ATP levels and inducing oxidative stress.^82^ Late, the inhibition of glycolysis and the PPP combined with the disruption of TRX system has proven to represent a successful strategy in selectively increase cytotoxicity in pancreatic and breast cancer cells but not in normal counterparts.^83^ These results suggest that a combined approach might be a better strategy in targeting malignant tumors when limited efficacy is observed with single agents.

### Fatty acid oxidation

The FAO is composed of a cyclical series of controlled oxidations that occur in the mitochondria of mammalian cells, through which long- and short-chain fatty acids are shortened, generating NADH, FADH2 (flavin adenine dinucleotide (reduced form) and acetyl-CoA to support biosynthetic pathways and produce ATP. However, in cancer cells, a consistent fraction of the acetyl-CoA enters into the tricarboxylic acid cycle (TCA) cycle and generates citrate, which is therefore exported into the cytosol and funneled into metabolic reactions catalyzed by the malic enzyme (ME) and the isocitrate dehydrogenase 1 (IDH1), that ultimately produce large amounts of NADPH.^84^ The importance of FAO for NADPH homeostasis and redox balance of cancer cells prevent cell death during loss of matrix adhesion^9^ and metabolic stress conditions through the modulation of the liver kinase B1 (LKB1)/AMP kinase axis.^85^ Overexpression of the key FAO regulators, such as the carnitine palmitoyltransferase-1,^86^ occurs in both solid tumors and leukemia cells,^87^ whereas its pharmacological inhibition by etomoxir was found to impair NADPH production and promote oxidative stress-induced cell death in human glioblastoma cells associated with profound ATP depletion^88^ and to strengthen the pro-apoptotic effect of cytotoxic agents in human leukemia cells.^89^ Given its importance in many types of tumor, targeting the FAO represent a promising novel strategy to disrupt the redox homeostasis of malignant cells and interfere with biosynthetic or bioenergetics processes that regulate cancer cell survival triggering either apoptosis-dependent or -independent cell death.

### Pentose phosphate pathway

The PPP is a major catabolic pathway of glucose through which cancer cells produce large amounts of ribose-5 phosphate, a precursor of nucleotide synthesis and NADPH, a key molecule that is used to drive anabolic processes and to detoxify harmful ROS.^90^ Activation of the PPP represents a key hallmark of many tumors where this metabolic pathway is found at the crossroad between glycolytic activity, unrestricted proliferation and scavenging of excessive ROS.^91^ The transcriptional regulation of glucose-6-phosphate dehydrogenase (G6PD), the rate-limiting enzyme of the PPP, by TAp73 and TAp63α was recently described in U2OS osteosarcoma cells, wherein its overexpression enhanced the PPP-dependent production of NADPH.^92, 93^ Additional mechanisms of G6PD regulation might directly depend on the availability of glucose: glucose funneling into the oxidative branch of the PPP directly controls the redox homeostasis of human clear cell carcinoma cells.^94^ This latest evidence underlines the importance of the PPP in the regulation of tumor cell survival and therapeutic resistance. In this respect, overexpression of G6PD promote doxorubicin resistance through increased GSH content and multidrug resistance-mediated efflux in HT29 colon carcinoma cells.^95^ In another study, the inactivation of the oncoprotein mucin1 C-terminal subunit restored the sensitivity of multiple myeloma cells to bortezomib, preventing the TIGAR-dependent glucose entry into the PPP and inducing massive ROS accumulation due to GSH depletion.^96^ Simultaneous inhibition of glycolysis and PPP through 2-deoxy-d-glucose and 6-aminonicotinamide, respectively, induced oxidative stress and sensitized malignant human cancer cell lines to radiotherapy presumably through the induction of multiple cell death modalities including apoptosis, necrosis and mitotic catastrophe.^97^ The functional inactivation of rate-limiting enzymes of the PPP or the hindrance of glucose funneling into the G6PD-dependent reactions could represent a promising strategy in overcoming intrinsic or acquired resistance to conventional chemo/radiotherapy in both solid and hematologic tumors.

### Glutaminolysis

Glutamine is a non-essential amino acid that has a key role in tumor metabolism, serving as a source of carbon and nitrogen for biosynthetic processes, an intermediate for energy production and a precursor for glutathione synthesis.^98^ Increased glutamine catabolism is a common hallmark of tumor metabolism reprogramming through which cancer cells support cell proliferation, signal transduction and redox homeostasis.^99^ It is generally assumed that the expression levels of certain oncogenes (i.e., Ras and Myc) or tumor suppressors (i.e., p53) can strongly influence the extent of glutamine utilization and the metabolic profile of different tumors.^5, 100, 101, 102^ However, emerging aspects of glutamine metabolism concern the potential mechanisms through which glutamine utilization can regulate the redox balance of malignant cells.^68, 103^ Indeed, it is increasingly recognized that glutaminase enzymes directly contribute to glutathione synthesis converting glutamine into glutamate and promoting the uptake of cysteine through the Slc7a11 exchanger.^104^ Similarly, metabolic intermediates such as citrate can be diverted from the TCA cycle and exported into the cytosol, where ME or IDH1 use them to generate reducing power in the form of NADPH.^84^ This strategy helps tumors to keep the glutathione pool in a reduced state and support the TRX system.^105, 106^ Additional mechanisms of redox modulation have been reported, wherein the mitochondrial enzyme glutamate dehydrogenase 1, by controlling the intracellular fumarate levels, positively regulates the enzymatic activity of the antioxidant enzyme glutathione peroxidase (Gpx).^11^ Given the paramount importance of glutamine metabolism in tumor progression and redox control, interfering with its function might represent an attractive anticancer strategy.^107^ With this respect, the glutaminase enzymes (GLS) as master regulators of glutamine metabolism have been the focus of recent anticancer research. Pharmacological inhibition of GLS1 with bis-2-(5-phenylacetamido-1,3,4-thiadiazol-2-yl)ethyl sulfide (BPTES) impaired the proliferation of P493 B-cell lymphoma (BCL) cells inducing DNA fragmentation and apoptotic cell death, whereas the genetic silencing of GLS1 prolonged the survival of mice with Myc-induced hepatocellular carcinoma and markedly impaired the growth of P493BCL xenograft.^108^ Similarly, BPTES-induced GLS1 inhibition selectively suppressed the growth of glioma cells with the R132D mutation in the IDH1 isoform^109^ and blocked the proliferation of primary acute myeloid leukemia (AML) cells with mutations in the IDH1/2 enzymes.^110^ By comparing different AML cell lines, Goto *et al.*^111^ have reported that glutaminolysis inhibition was associated to the depletion of the intracellular GSH content and subsequent ROS generation, particularly in HL-60 cells characterized by glutamine addiction. In a former study, glutamine deprivation also decrease the GSH levels of neuroblastoma cells, leading to altered redox balance, impaired cell proliferation and increased chemosensitivity to the alkylating agent L-PAM.^112^ Finally, the combined inhibition of GLS1 and heat-shock protein 90 induced synthetic lethality in cancer cells and mouse embryonic fibroblasts with activating alterations of the mTORC1 pathway through increased ER stress and disruption of the redox balance caused by GSH depletion.^83^ Taken together, these studies underline the importance of glutamine in the maintenance of redox balance, cell growth and cell survival of both solid and hematologic tumors, setting the rational for therapeutic approaches aimed at the manipulation of glutamine metabolism to block malignancy.

### The serine–glycine one-carbon metabolism

The serine–glycine one-carbon metabolism (SGOC) is a complex network of biochemical reactions that integrate inputs from amino acids and glucose derivatives (mainly serine and glycine) and generates multiple outputs as carbon units (tetrahydrofolate (THF) and its derivate) that serve different cellular functions. The redistribution of these carbon units from serine and glycine rely on three pathways: the folate cycle, the methionine cycle and the *trans*-sulfuration pathway.^113^ Folate, a vitamin B derivative, is reduced to THF by a series of metabolic reactions and converted into methylenetetrahydrofolate by serine hydroxymethyl transferase (SHMT). This product is either converted to F-THF or reduced by methylenetetrahydrofolate reductase to methylenetetrahydrofolate, whose demethylation completes the folate cycle. The carbon units therefore enter into the methionine cycle with the generation of *S*-adenosylmethionine by the methionine adenyltransferase, with further conversion by *S*-adenosyl homocysteine hydrolase into homocysteine.^114^ The last modular component of the one-carbon metabolism, the *trans*-sulfuration pathway, is functionally connected to the methionine cycle through the homocysteine, whose condensation with serine by cystathionine synthase generates cystathione, further metabolized to alpha-ketobutyrate and cysteine by cystathione lyase. The cysteine can therefore be diverted into GSH synthesis.^113^ For long time, SGOC has been associated with cancer cell due to its importance for the regulation of nucleic acid, lipids and protein synthesis of proliferating cells. More recent evidence indicates that this pathway is also crucial for redox balance.^115, 116^ With this respect, the mitochondria have been shown to have a prominent role.^117^ Indeed, despite THF-derived carbon units are primarily used for nucleotide synthesis in the cytosol, new methods for tracing NAPDH compartmentalization indicate that serine is predominantly utilized in the mitochondria of mammalian cells to generate NADPH.^67, 118^ An observation that was further substantiated by a recent study showing that both glycine and serine catabolism were responsible of NADPH production in the mitochondria of HEK-293 and MDA-MB468 cell lines.^67^ Interestingly, Myc-transformed cells subdued to hypoxia strongly upregulated the expression of the mitochondrial SHMT2, responsible for an abundant NADPH production. Conversely, knockdown of SHMT2 impaired antioxidant ability and increased cell death under hypoxia, but by a not yet known mechanism.^119^ Also, the antioxidant transcription factor Nrf2 can regulate the expression of key one-carbon metabolism enzymes, including 3-phosphoglycerate dehydrogenase, phosphoserine aminotransferase 1 and methylenetetrahydrofolate dehydrogenase 2 (MTHFD2) in human non-small cell lung cancer (NSCLC) cells, supporting nucleotide and glutathione synthesis.^120^ Despite the fact that further studies will be necessary to assess potential therapeutic benefit of this approach, novel and selective inhibitors of SHMT2 and MTHFD2 enzymes might represent a promising anticancer strategy against hypoxic tumors characterized by otherwise limited tractability.^117^ Remarkably, the use of antifolate such as methotrexate and pemetrexed, which are known inhibitors of active SHMTD, still represents a cornerstone of antineoplastic therapy against solid and hematologic tumors including breast cancer, bladder cancer, acute lymphoblastic leukemia and lymphomas.^121^ Also, in therapy-resistant tumors, downstream pathways of one-carbon metabolism have been successfully targeted with agents that interfere with the nucleotide synthesis such as 5-FU for advanced colorectal cancer or gemcitabine for pancreatic cancer.^113^ Given the increased interest in metabolic alterations of cancer cells and the intimate connection between SGOC pathways and redox homeostasis of human tumors, key nodes in the one-carbon metabolism might represent a valid therapeutic target at the crossroad between the regulation of cancer growth and antioxidant capacity.

## Mitochondria: The Perfect Location to Target Redox Homeostasis and Metabolic Pathways

One emerging aspect in the study of molecular mechanisms controlling redox balance and metabolism in mammalian cells regards the existence of a clear compartmentalization of specific biochemical reactions in different cell organelles. It is increasingly known that mitochondria are key organelles for the regulation of redox signaling and redox homeostasis of normal and cancer cells (Figure 1). By integrating metabolic, bioenergetics and redox cues, the mitochondrial network acts as a central hub that directly or indirectly controls a wide number of cellular processes including proliferation, ATP synthesis and cell death.^122^ By hosting multiple redox-active complexes and metabolic enzymes that generate superoxide anion, the mitochondria represent a major source of endogenous ROS production. The most well-characterized site is represented by the mETC, through which the electrons from reduced metabolic intermediates (e.g., NADH and FADH2) are transferred to the molecular oxygen. During the electrons flow, depending on the mitochondrial membrane potential status and the oxygen availability, semiquinone radicals can be generated at the level of complexes I, II and III, promoting the univalent reduction of oxygen into superoxide.^123^ Other sources of mitochondrial-dependent superoxide production include the 2-oxoglutarate dehydrogenase, the pyruvate dehydrogenase in the mitochondrial matrix, the mitochondrial glycerol-3 phosphate dehydrogenase and the electron transfer flavoprotein-ubiquinone oxidoreductase mitochondrial system (flavoprotein-ubiquinone oxidoreductase) located in the inner membrane.^38^ The generated superoxide can then leave the mitochondrial district through different ways. One well-established route is through its conversion into H_2_O_2_ by SOD2, whereas the second mechanism, still a matter of intense debate, postulates its direct diffusion into the cytosol through the voltage-dependent anion channel (VDAC),^124, 125^ wherein is spontaneously or enzymatically transformed into H_2_O_2_ by SOD1. In this way, the mitochondria produce picomolar to nanomolar amounts of peroxide that then leave the site of generation and promote retrograde signaling to the nucleus or regulate activity, localization and stability of redox-sensitive target proteins that are located in the cytosol.^126^ On the basis of their central role in redox control and metabolism, the mitochondria represent attractive targets for anticancer therapy. Several studies have investigated the effect of mitochondrial-targeted antioxidants and their impact in tumor biology.^127^ MitoQ is an orally active antioxidant that not only mimics the role of the endogenous mitochondrial antioxidant coenzyme Q10 (CoQ10) but also substantially augments the antioxidant capacity of CoQ10 in a mitochondrial membrane potential-dependent manner.^128^ MitoQ has been found to kill breast cancer cells and unhealthy mammary cells, supporting a role for MitoQ and similar compounds to be further evaluated for novel anticancer activity.^129^ Among other aspects, the relative contribution of different metabolic pathways involved in NADPH generation (e.g., ME1, IDH1, PPP and glutaminolysis) has received significant attention^1, 102^ with particular emphasis on the role of one-carbon metabolism.^113^ With this respect, mitochondrial NADPH mainly derives from serine catabolism regulated by SHMT2 and MTHFD2, two mitochondrial-specific enzymes that are frequently overexpressed in cancer cells acting as important regulators of tumor redox homeostasis, but absent or underrepresented in normal tissues.^67, 130^ Genetic depletion of SHMT2 altered the redox balance of Myc-transformed tumors during hypoxia and induced significant cytotoxicity,^119^ whereas knockdown of MTHFD2 in overexpressing breast cancer tumors has been shown to impair cell migration and invasion, and to sensitize malignant cells to methotrexate by inducing caspase 3/7-independent cell death.^131^ Taken together, these observations imply that targeting multiple mitochondrial functions with single agents or in combination with inhibitors of different metabolic pathways might represent a promising approach to improve the efficacy of conventional chemotherapeutics, in particular in those tumors wherein the apoptotic machinery is not functional.

Despite the fact that mitochondrial dysfunction has long been considered a metabolic hallmark of cancer cells, studies have also indicated that tumor cells not only have functional mitochondria but also that their activity is essential for tumorigenesis.^127^ By regulating the generation of ROS, ATP and other metabolites driving bioenergetic and biosynthetic processes, mitochondria have a key role in cancer progression.^132, 133^ Oncogenic activation of MYC and KRAS promote increased glucose utilization and addiction in different types of tumors,^134^ while also enhancing the mitochondrial-dependent biosynthesis of macromolecules through increased ATP levels and TCA cycle intermediates.^64, 135^ To replenish the TCA cycle substrates and fuel their uncontrolled growth, cancer cells utilize specific mechanisms of anaplerosis on the basis of the oxidation of glutamine to α-ketoglutarate and its conversion to oxaloacetate.^136^ As a consequence of oxidative metabolism, physiological amounts of ROS are produced at the level of the mETC, inducing the pro-tumoral activation of redox-sensitive pathways.^31^ To prevent ROS-induced toxicity, cancer cells redirect the metabolic intermediates coming from glutamine and one-carbon metabolism into alternative pathways that generate NADPH and GSH, antioxidants molecules readily used by several ROS-scavenging enzymes.^67, 118^ Interestingly, tumors with impaired TCA cycle activity (or mutations in the mETC complexes) and that rely on glycolysis for ATP synthesis, shift to reductive glutaminolysis to mediate biosynthetic processes and cell survival.^137, 138^ Such tumor cells depend on mitochondrial-derived ROS to promote cell proliferation and metastasis formation.^139,140,141^ In contrast to what is generally assumed, many tumors still use mitochondria for ATP production, despite the Warburg effect should provide sufficient amounts of ATP for their biological needs.^142^ Indeed, poorly vascularized and other subsets of tumors growing under limited glucose conditions heavily rely on oxidative phosphorylation for ATP synthesis,^87, 143, 144^ a weakness that might be targeted with drugs that limit glucose utilization and block mitochondrial bioenergetics.^145, 146^ With this respect, two anti-diabetic biguanides, such as metformin and phenformin, show promising antitumoral effects.^147^ Epidemiological studies have shown that metformin decreases the incidence of cancer and prolongs the survival rate of patients with solid tumors.^148, 149^ More studies have reported also that the *in vivo* antitumorigenic effects of metformin depended on the inhibition of the mETC complex-I activity and the decrease of circulating glucose and insulin levels.^150,151,152^ Metformin was also able to impair the growth of NSCLCs by blocking the activation of the Akt/mammalian target of rapamycin (mTOR) pathway and by potentiating the pro-apoptotic efficacy of ionizing radiation.^153^ Also, metformin selectively killed breast cancer stem cells through profound depletion of triphosphate nucleotides presumably reflecting a major impairment of the energetic metabolism.^154^ Therefore, another biguanide, called phenformin, has emerged as a potential anticancer agent due to its liposolubility, higher affinity for mETC complex-I and stronger antineoplastic activity.^155^ Phenformin enhanced the efficacy of the BRAF inhibitor PLX4270 both *in vitro* and in genetic models of melanoma driven by BRAF^V600E^ mutations triggering apoptotic cell death upon inhibition of the mTOR pathway.^156^ Phenformin also induced selective apoptosis in a subset of NSCLCs with loss of the tumor suppressor LKB1 and oncogenic mutations in K-ras lowering the ATP levels and eliciting the activation of caspase 3.^157^ Despite the encouraging results, it should also be mentioned that the use of phenformin has been associated with lactic acidosis, an important pitfall that might limit its clinical applicability.^158^ For this reason, novel compounds that target the mETC complex-I (i.e., VLX600) or alternative strategies based on the translation of specific mitochondrial proteins (i.e., tigecycline) have been designed and currently successfully employed in preclinical studies.^77, 159^ Although the potential utility of these compounds still needs to be validated in long-term clinical trials, encouraging evidences suggest that targeting the bioenergetics function of the mitochondria might represent a valid therapeutic option for cancer treatment.

## Conclusions and Perspective

**Figure 3:**
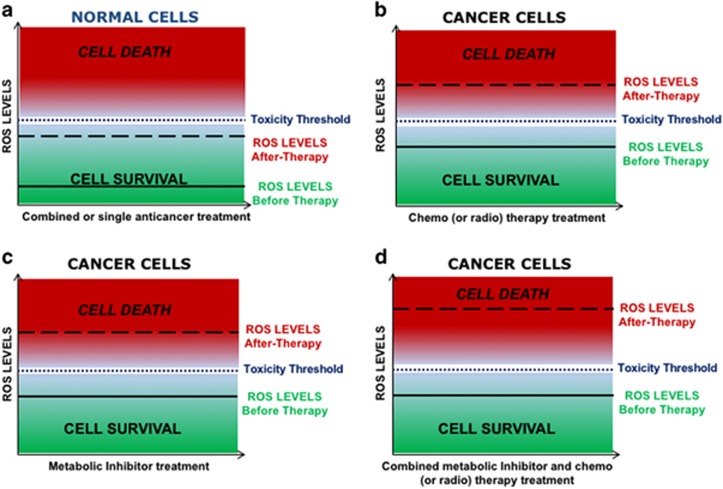
Strategies to manipulate ROS levels as anticancer therapy. Effect of different therapeutic manipulations on the intracellular ROS levels and relative toxicity in both normal and cancer cells. (**a**) Normal cells treated with conventional chemo/radiotherapy, metabolic inhibitors or combined therapy show a slight increase in cell death. On the contrary treatment of cancer cells with (**b**) chemo/radiotherapy or (**c**) metabolic inhibitors elevates the rate of cell death compared with normal cells due to higher basal levels of ROS. When combined approaches on the basis of the use of metabolic inhibitors and conventional therapy (**d**) or other ROS-inducing agents can synergistically eradicate a larger proportion of cancer cells with marginal impact on normal cells, by elevating the intracellular ROS levels far above the toxicity threshold

In light of recent research, the inhibition of metabolic pathways or ROS-scavenging mechanisms, followed by the administration of pro-oxidizing agents (i.e. chemo-radiotherapy), represents a promising therapeutic option for tumors characterized by resistance to treatment (Figure 3). With this respect, it is well documented that the efficacy of many anticancer therapies, including chemo- and radiotherapy, largely depends on the extent of the evoked ROS production.^14^ In many studies, either metabolic inhibition or the blockade of certain antioxidant systems was shown to strongly sensitize cancer cells to apoptotic cell death induced by further elevation of ROS levels.^160, 161, 162, 163, 164^ However, due to the undesired side effects including cardio-toxicity and nephrotoxicity, extensive research has been pursued to identify novel ROS modulators with a safer therapeutic profile. With this respect, another class of quinone-based compounds, including menadione and other vitamin K3 derivatives, are emerging as promising anticancer agents.^165^ Remarkably, this combinatorial approach has the ability to engage multiple cell death modalities, not limited by the activation of the intrinsic and extrinsic apoptotic pathways, and therefore might be useful to overcome the mechanisms of therapy resistance due to the overexpression of anti-apoptotic proteins or compromised activation of the caspase cascades. With this respect, the induction of ferroptosis, an iron- and ROS-dependent form of non-apoptotic cell death characterized by altered mitochondrial morphology, is receiving increasing attention for its intimate connection with cellular metabolism and redox balance.^166, 167^ Indeed, large B lymphomas and renal cell carcinomas with Ras mutations were found to be particularly susceptible to ferroptosis upon BSO-mediated GSH depletion, decreased Gpx4 activity and accumulation of lipid peroxidation.^168, 169^ In another study erastin, a potent inhibitor of X_c_^−^ cystine importer was shown to induce ER stress and trigger ferroptosis in different cancer cell lines.^170^

In conclusion, it is becoming clear that redox signaling events require the simultaneous regulation of sources, transducers and scavengers in a precise spatial and temporal framework, whose alteration may disrupt key redox nodes and promote aberrant signaling. The loss of control over specific redox circuitries is likely to represent a tumor-specific alteration that may trigger unrestricted proliferation and malignant progression. Moreover, metabolomics studies associated to computational models on the basis of the integrative bioinformatics are becoming increasingly accessible and will surely guarantee a rapid progress in the field of redox biology, in particular addressing how metabolism and its subcellular compartmentalization can influence ROS signaling and redox cofactors. We are now approaching a new era wherein ROS biology and their effects in the physiopathology of cancer may be dissected with unprecedented detail, bringing potential therapeutic benefits derived from selective manipulations of cancer redox balance to be uncovered, paving the way to novel and exciting investigations in the fight against cancer.

## Figures and Tables

**Table 1:** Metabolic blockade-based anticancer treatments and their effect on metabolism or redox balance

**Name**	**Type of tumor**	**Mechanism of action**	**Impact on redox and metabolism**	**Clinical stage**	**References**
Etomoxir	Glioblastoma; leukemia	Inhibition of CPT1	ROS elevation through NADPH and ATP depletion	Preclinical	^89^
6-aminonicotinamide	Prostate cancer; head and neck carcinoma	Inhibition of 6-phosphogluconate dehydrogenase	ROS increase through NADPH and GSH decrease	Approved	^94, 97^
Buthionine sulfoximine	Breast cancer; acute limphoblastic leukemia; multiple myeloma	Inhibition of GSH neosynthesis mediated by glutamate cysteine ligase	ROS increase due to GSH depletion	Approved	^163, 168, 169^
Bis-2-(5-phenylacetamido-1,3,4-thiadiazol-2-yl)ethyl sulfide; compound 968	B-cell lymphoma; acute myeloid leukemia; pancreatic cancer	Inhibition of glutaminase enzymes	Decreased intracellular GSH content and enhanced sensitivity to pro-oxidants	Approved	^108,109,110^
Arsenic trioxide	Small cell lung cancer; hepatocellular carcinoma; acute promyelocytic leukemia	Inhibition of mitochondrial respiration; cross-linking of thiols in redox-sensitive cysteines of GSH and antioxidant enzymes	Increased susceptibility to oxidative stress due to GSH oxidation and inactivation of Trx1, Trx2, Prx3 and Gpx2; decrease in ATP synthesis due to factor B inhibition	FDA approved	^22, 161, 163, 164^
Anthracyclines (doxorubicin, daunorubicin)	Colon carcinoma; breast cancer; neuroblastoma	Redox cycling and *S*-glutathionylation of mETC proteins; alteration of iron homeostasis	Increased ROS production due to Fenton's reaction and inhibition of the mETC complexes	FDA approved	^95^
Cisplatin	Non-small lung cancer; ovarian cancer;	Interference with the mETC activity; activation of the NADPH oxidases	Induction of intracellular and mitochondrial ROS production leading to lipid peroxidation, DNA damage and Ca^2+^ influx	FDA approved	^48,49,50,164^
Menadione	Pancreatic carcinoma; lung cancer	Triggering of redox cycling reactions; arylation of cellular thiols provoking GSH depletion	Increased ROS levels due to redox cycling; increased susceptibility to oxidation due to GSH decrease	Phase II	^165^
Metformin; phenformin	Melanoma; breast cancer; non-small lung cancer	Interference with mETC activity	Increased mitochondrial ROS production	Phase 0–II	^148, 149,150,153,154,155,156,157,158^

Abbreviation: CPT1, carnitine palmitoyltransferase-1

## Abbreviations

AML: acute myeloid leukemia
ATP: adenosine tris-phosphate
BCL: B-cell lymphoma
BPTES: bis-2-(5-phenylacetamido-1,3,4-thiadiazol-2-yl)ethyl sulfide
FADH2: flavin adenine dinucleotide (reduced form)
FAO: fatty acid oxidation
G6PD: glucose-6-phosphate dehydrogenase
GDH1: glutamate dehydrogenase 1
GLS1: glutaminase 1
GSH: reduced glutathione
H_2_O_2_: hydrogen peroxide
IDH1,2: isocitrate dehydrogenase 1,2
LDH: lactate dehydrogenase
ME: malic enzyme
mETC: mitochondrial electron transport chain
MTHFD2: methylenetetrahydrofolate dehydrogenase 2
MTOR: mammalian target of rapamycin
NADH: nicotinamide adenine dinucleotide (reduced form)
NADPH: nicotinamide adenine dinucleotide phosphate
NSCLC: non-small cell lung cancer cell
PPP: penthose phosphate pathway
ROS: reactive oxygen species
SGOC: serine–glycine one-carbon metabolism
SHMT2: serine hydroxymethyl transferase 2
THF: tetrahydrofolate
TRX: thioredoxin
